# Analysis of chimera states as drive-response systems

**DOI:** 10.1038/s41598-018-20323-2

**Published:** 2018-01-30

**Authors:** André E. Botha, Mohammad R. Kolahchi

**Affiliations:** 1 Department of Physics, University of South Africa, Private Bag X6, Florida, 1710 South Africa; 2 Department of Physics, Institute for Advanced Studies in Basic Sciences, Zanjan, 45195-1159 Iran

## Abstract

Chimera states are spatiotemporal segregations – stably coexisting coherent and incoherent groups – that can occur in systems of identical phase oscillators. Here we demonstrate that this remarkable phenomenon can also be understood in terms of Pecora and Carroll’s drive-response theory. By calculating the conditional Lyapunov exponents, we show that the incoherent group acts to synchronize the coherent group; the latter playing the role of a response. We also compare the distributions of finite-time conditional Lyapunov exponents to the characteristic distribution that was reported previously for chimera states. The present analysis provides a unifying explanation of the inherently frustrated dynamics that gives rise to chimera states.

## Introduction

In the classic chimera state, a set of identical and identically coupled oscillators becomes divided into two coexisting, interdependent groups: one moving coherently, in the sense that the average frequencies of the oscillators are the same, the other moving incoherently^1^. Rather than emerging via spontaneous symmetry breaking, the chimera typically evolves from certain symmetry-broken initial conditions^2,3^. Once established, however, it can persist indefinitely, even for finite numbers of oscillators^4^. Other than being of theoretical interest, chimera states have been observed in a wide variety of experimental settings. See, for example, the introduction of ref.^3^, and the references given therein. For a recent review of chimera states, see Panaggio and Abrams^5^.

Since the discovery of chimera states, more than fifteen years ago^6^, much attention has been drawn to the fact that the two sectors appear to cooperate in sustaining the overall chimera character of the state. However, despite numerous studies, relatively little progress has been made towards explaining this mutually sustaining nature of the two groups.

The term, frustration, familiar from equilibrium studies^7^, has also been used in connection with the Kuramoto-Sakaguchi model^8^ (or frustrated Kuramoto model^9^), where a global phase shift *α*, called the frustration parameter, is present in the sine coupling function. In the past, Lyapunov functions have been used to study the dynamics in the original Kuramoto model^10^, with both attractive^11^ and repulsive^12^ coupling. The presence of *α*, however, precludes the possibility of defining a Lyapunov function for the system, thus making its analysis in terms of an energy landscape more complicated. Curiously, the literature on chimera states exclusively refers to *α* as the phase lag^5^. The reason for this change in nomenclature is unclear. In the article by Panaggio and Abrams^5^, for example, the effect of *α* on the dynamics is suggested as one of several open questions about chimera states. Here, we take up the familiar notion of frustration, but now in a dynamic sense. As in previous studies^13–17^, we make use of the nonlocally coupled Kuramoto-Sakaguchi model, which has been shown to support stable chimera states, even for relatively small numbers of oscillators. See, for example, Fig. 5 of ref.^4^, for the case *N* = 30, at *r* = 0.

Recently, Andrzejak *et al*.^17^ investigated the possibility of synchronizing chimera states across different interacting networks in which the individual networks were capable of sustaining chimera states. They found that generalized synchronization^18^ could occur between two non-identical networks, containing the same number of oscillators, at two different values of *α*. For generalized synchronization to occur, nodes in the driver network had to be coupled unidirectionally to nodes in the response network^19^. The discovery of generalized synchronization^18^ and phase synchronization^20^ have greatly extended the applicability of dynamical systems theory to chaos synchronization in general and, in the aforementioned study by Andrzejak *et al*.^17^, to chimera states. However, generalized synchronization of chaos cannot be used to model individual systems in which chimera states occur. This is because the nonlocal coupling, in the typical systems that support chimera states, is not unidirectional. To develop a model of chimera states, we therefore propose the framework contained in the influential paper by Pecora and Carroll^21^, on the synchronization of chaotic systems.

Pecora and Carroll^21^ were the first to investigate chaos synchronization between two identical dynamical systems in which arbitrary subsets of variables from one of the systems, called the drive, were used to replace the corresponding variables in the other, called the response. They discovered a purely dynamic criterion for the synchronization of the two chaotic systems; namely, that the sub-Lyapunov exponents^21^, later termed the conditional Lyapunov exponents^22^, should all be negative.

In the present work we apply Pecora and Carroll’s theory to obtain insights into the inner workings of chimera states. We show that the division of the coupled system of identical oscillators, into the incoherent and coherent groups, corresponds to Pecora and Carroll’s drive-response system. The incoherent group acts like a drive which maintains the order within the coherent group. In this way the inherently frustrated nature of the chimera is revealed. We also show that the present interpretation is consistent with that given previously for the peaks of the characteristic distribution of finite-time Lyapunov exponents for chimera states^16^.

## Results

As in the work by Pecora and Carroll^21^, we start with two ‘identical’ systems. In our case, each system is a nonlocally coupled Kuramoto-Sakaguchi model^13^:

1 $${\dot{\phi }}_{i}=-\frac{K}{2R}\sum _{j=-R}^{R}\,\sin ({\phi }_{i}-{\phi }_{i+j}+\alpha ),$$

2 $${\dot{\theta }}_{k}=-\frac{K}{2R}\,\sum _{j=-R}^{R}\,\sin ({\theta }_{k}-{\theta }_{k+j}+\alpha ).$$

Here *i*, *k* = 1, 2, …, *N*, the integer *R* quantifies the coupling range, and *K* controls the coupling strength.

The way the system is divided into a drive and a response is crucial to its stability and synchronization properties^21^. In what follows we show how this subdivision is related to the structure of the chimera. Following Pecora and Carroll^21^, we wish to substitute some of the variables from the drive system (1), in place of the corresponding variables in the response (2). At first, such a substitution may appear to be contrary to the notion of the chimera state; since, one might object, when some of the driving *φ*_*k*_ are substituted for the corresponding *θ*_*k*_, all the oscillators in the response system (2), are no longer identical – because some are now being driven. While this loss of equivalence among the oscillators does break the symmetry between the driving and response system^17^, it is important to realize that the driving in no way affects the identicalness of the oscillators within the drive itself. Thus, the drive can still support a true chimera state; even though the response system no longer strictly fulfills the identicalness requirement. Crucially, however, Pecora and Carroll’s conditional Lyapunov exponents depend only on the dynamics of the drive, and thus the prediction they make about the chaos synchronization between the two ‘identical’ systems, are in fact predictions about the behavior of one and the same system; namely, the unaltered ‘drive’ system.

To clarify this point further, we summarize the main equations from Pecora and Carroll’s original paper^21^, in which they subdivide an *N*-dimensional autonomous system, written symbolically as

3 $$\dot{u}=f(u),$$

into two subsystems:

4 $$\dot{v}=g(v,w),\,{\rm{and}}\,\dot{w}=h(v,w),$$

with *v* = (*u*_1_, *u*_2_, …, *u*_*m*_), *w* = (*u*_*m*+1_, *u*_*m*+2_, …, *u*_*N*_), *g* = (*f*_1_(*u*), *f*_2_(*u*), …, *f*_*m*_(*u*)), *h* = (*f*_*m*+1_(*u*), *f*_*m*+2_(*u*), …, *f*_*N*_(*u*)), and the response system given by $\dot{w}^{\prime} =h(v,w^{\prime} )$. Notice that the response system (primed variables) is being driven by a subset of dynamical variables, *v*, taken from the ‘drive’ system (3). The variational equation, determining the synchronization between *w* and *w*′, is then found to be of the form

5 $$\dot{\xi }={D}_{w}h(v(t),w(t))\xi ,$$

where, “*D*_*w*_*h* is the Jacobian of the *w* subsystem vector field with respect to *w* only”^21^. The conditional Lyapunov exponents are thus simply projections of the dynamics onto an appropriate subspace – they measure the growth or contraction of the principle axes (frame vectors) defined by the linearization of drive system, restricted to the *w* subspace; i.e., to the subspace spanned by the dynamical variables which are not directly participating in the driving. As such, the concept of conditional (or sub-) Lyapunov exponents can be extended to any *N*-dimensional autonomous system, even if no driving actually occurs.

With the previous paragraph as motivation, we follow Pecora and Carroll’s approach by setting, in Eq. (2), ${\theta }_{\ell }={\phi }_{\ell }$ for $\ell \in [(N-M+2)\mathrm{/2},(N+M\mathrm{)/2}]$, where *M* is the number of drive oscillators. For simplicity of notation, and without any loss of generality, we assume here that *N* and *M* are even numbers, with *M* = 0 corresponding to the case of no driving. A description of the numerical procedure followed to solve Eqs (1) and (2), is provided in the first subsection of **Methods**. Details of the numerical procedures which we used to calculate both the long-time averaged and finite-time conditional Lyapunov exponents, are given in the second subsection of **Methods**.

**Figure 1:**
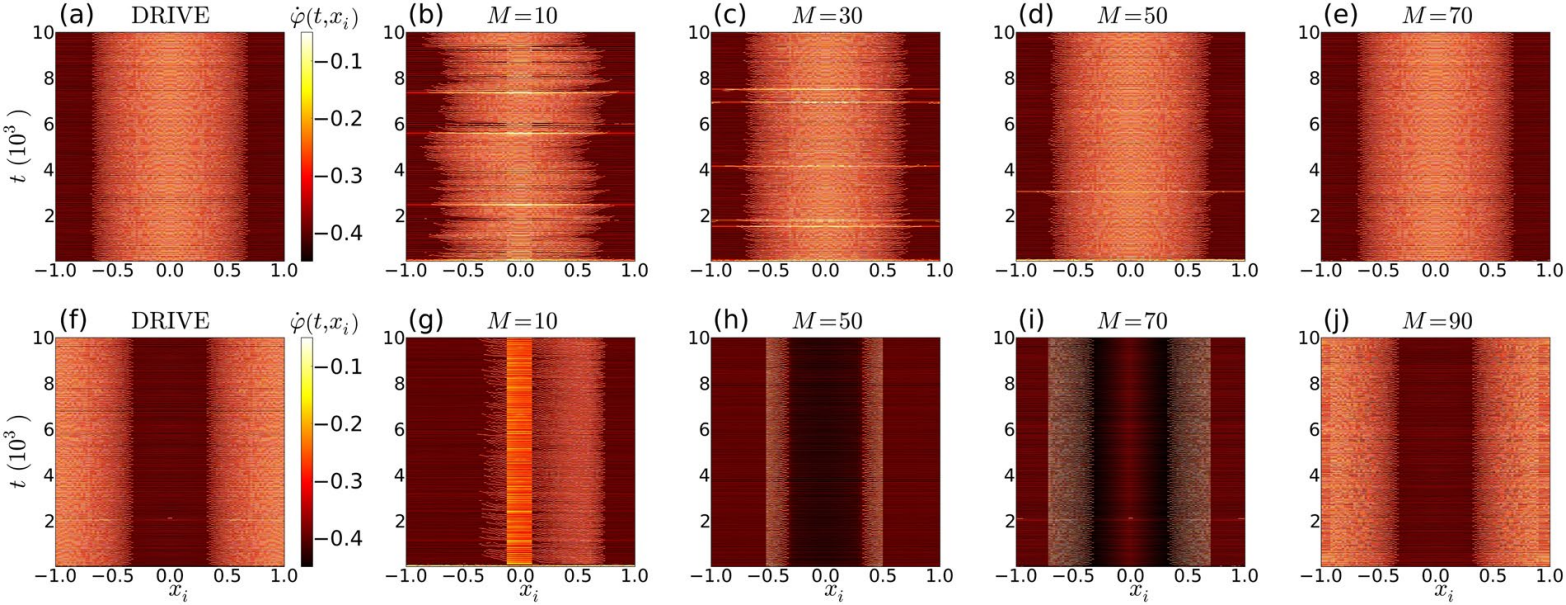
Time evolution of the instantaneous phase velocities. The panels show $\dot{\phi }(t,{x}_{i})$ for the two systems of oscillators in Eqs (1) and (2), with *N* = 100, *K* = 1, *R* = 0.35 *N* and *α* = 1.47. (**a**) and (**f**) show the drive system (1), prepared in symmetrical chimera states. In (**a**) the lighter coloured (incoherent region), and in (**f**), the darker coloured (coherent region), has been centred on *x* = 0. In (**b**–**e**) the response corresponding to (**a**) is shown for increasing numbers *M* of drive oscillators. The drive oscillators are positioned symmetrically about the centre of the incoherent region (*x* = 0). Full synchronization of the systems in (**a**–**e**) only occurs when all the incoherent oscillators (or more) are driving the response system, i.e. for *M* > 70. For the coherent centred drive shown in (**f**), the response is shown in (**g**–**j**). In this case the two systems, i.e. the coherent centred drive and the response, never fully synchronize, even at very large values of *M*. Their failure to synchronize can be seen by comparing the time evolution of the phase velocities for the coherent drive, as shown in (**f**), with those of the responses at the various values of *M*, as shown in (**g**–**j**).

Figure 1 summarizes our findings on the synchronization properties for different subdivisions. Both drive systems, shown in (a) and (f), are in chimera states. In (a)–(e) successively more drive oscillators were symmetrically added, starting from the centre of the incoherent region. In this case we see that the two systems fully synchronize when the drive consists of all the oscillators in the incoherent region of the chimera. On the other hand, when the drive oscillators are taken from the coherent region of the driving chimera, as in (f)–(j), full synchronization of the two systems only occurs trivially when *M* = *N*. For the coherent drive, even with *M* = 98, we found that the time averaged quantities, ${\langle {\dot{\phi }}_{1}-{\dot{\theta }}_{1}\rangle }_{t}$ and ${\langle {\dot{\phi }}_{100}-{\dot{\theta }}_{100}\rangle }_{t}$, differ significantly from zero – they are typically in the range [10^−4^, 10^−3^]. On the other hand, for the incoherent drive, with *M* ≥ 70, the differences, either averaged, or instantaneously measured, are on the order of the numerical accuracy, i.e. [10^−14^, 10^−13^]. We emphasize that we have tested both the instantaneous, and time averaged differences between the drive and response systems, because the coherent group is defined by adjacent oscillators that have equal time-averaged frequencies. Thus it is conceivable that the two systems could synchronize in a time-averaged sense, even though they are not fully synchronized.

The results in Fig. 1 suggest that, in the chimera state, the incoherent oscillators play the role of a drive, which acts to maintain the order in the coherent group. To explore this idea, we make use of Pecora and Carroll’s theorem^21^: the systems will only synchronize if the conditional Lyapunov exponents^22,23^ are all negative. In our case the conditional Lyapunov exponents are obtained from the integration of Eq. (1), together with its linearization, restricted to the subspace of dynamical variables that are not being used to drive Eq. (2).

**Figure 2:**
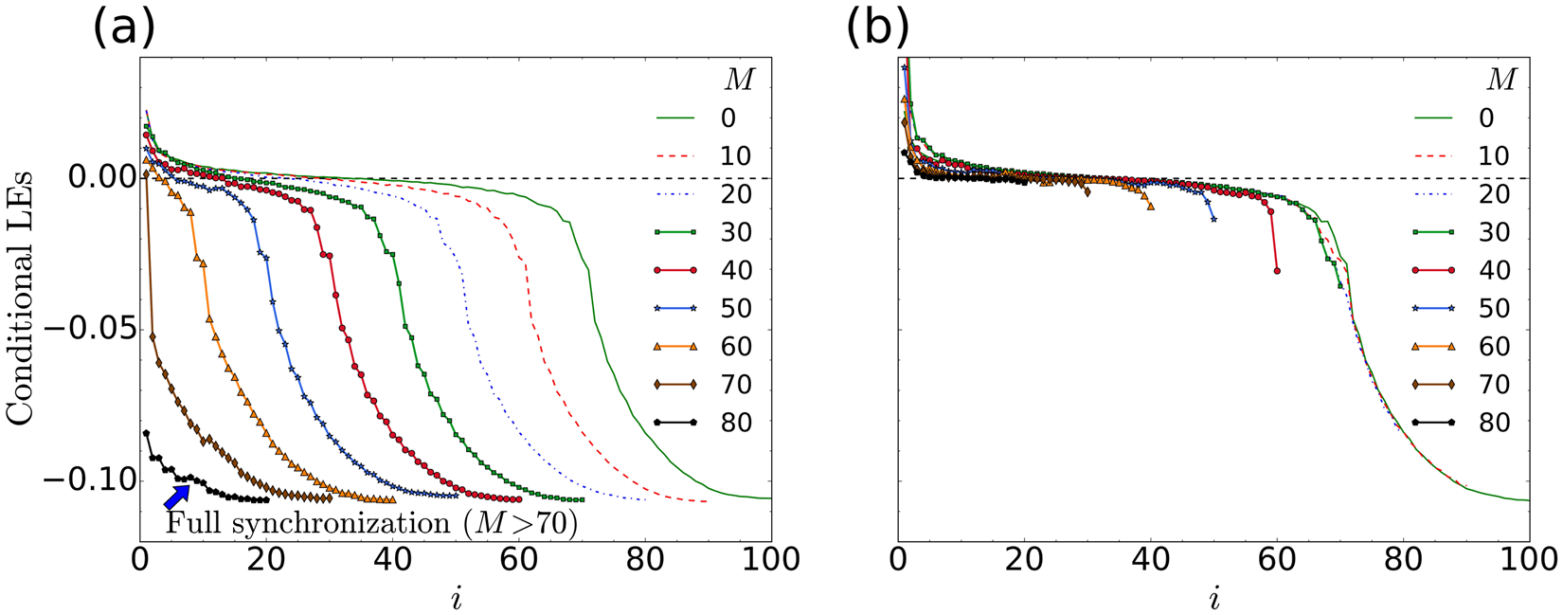
Conditional Lyapunov exponents, ordered from large to small. All parameters are the same as is Fig. 1. For comparison the ordinary Lyapunov exponents (in the case of *M* = 0), are also shown. Panel (a) is for incoherent-centred driving, as in Fig. 1(a). Panel (b) is for coherent-centred driving, as in Fig. 1(f). In (**a**) we see that there are some positive Lyapunov exponents for driving up to *M* = 70. Full synchronization only occurs for *M* > 70, when all the conditional Lyapunov exponents become negative. On the other hand, for the coherent-centred driving, in (**b**), we see that some of the conditional Lyapunov exponents remain positive, even for *M* ≥ 80. In this case the two systems never fully synchronize.

**Figure 3:**
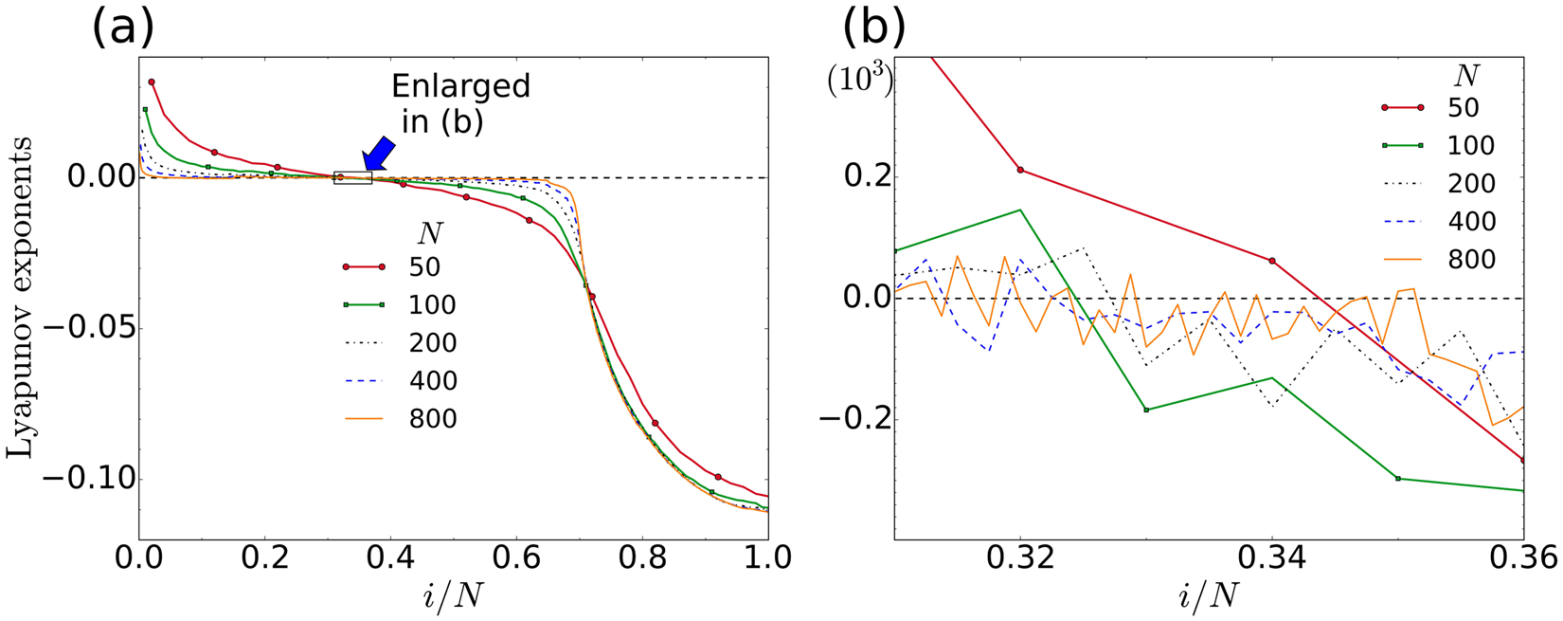
Lyapunov exponents, ordered from large to small. Panel (a) shows the Lyapunov exponents for chimera states of Eq. (1), at different values of *N*. All other parameters are the same as in Figs 1 and 2, and the Lyapunov exponents were averaged as before. Here, there is no driving and the chimeras are asymmetric in all five cases. (**b**) Enlarged view of the boxed region in (**a**), where the Lyapunov exponents cross from positive to negative. We see that the ratios of the number of positive Lyapunov exponents, to the total number of Lyapunov exponents, falls within a relatively narrow range [0.31, 0.36] of *i*/*N*. The small differences seen in the crossing ratios correspond to different *N*_I_/*N* ratios for the chimeras at various *N*. The *N*_I_/*N* ratios differ due to the different initial conditions used in each simulation. Hence, the crossing ratios are essentially independent of *N*.

In Fig. 2 we show the conditional Lyapunov exponents for the two different driving configurations; i.e. for the incoherent and coherent drives that were seen previously in Fig. 1(a,f), respectively. With the view of comparing Fig. 2, to Fig. 1, let *N*_I_ and *N*_C_ denote the number of incoherent and coherent oscillators in the chimera state, with *N* = *N*_I_ + *N*_C_. Our results indicate that, at fixed parameters, the ratio *N*_I_/*N* is independent of *N*, though it depends, unpredictably, on the exact initial condition that was used to set up the chimera. For a description of the initial conditions, see Abrams and Strogatz^1^. Furthermore, we found that *N*_I_ is approximately equal to the number of negative ordinary (i.e. not conditional) Lyapunov exponents. An example of this rule can be seen in Fig. 2, for the *M* = 0 cases, for which there are approximately 68 negative Lyapunov exponents. This value can be read off more clearly from the *N* = 100 curve (given by the green line), in Fig. 3(b). On the other hand, by counting the incoherent oscillators in Fig. 1(a) or (f), we find that *N*_I_ ≈ 70.

The ratio *N*_I_/*N* is insensitive to the system size. In Fig. 3, we have calculated the Lyapunov exponent spectra corresponding to the *M* = 0 cases in Figs 1 and 2, for *N* = 50, 100, 200, 400 and 800. Our analysis suggests that the formation of the two regions in a chimera state may be a natural way for the system to overcome its inherent dynamic frustration. In the language of Pecora and Carroll’s pioneering work^21,22^, the chimera coherent-driving-incoherent subsystem is unstable and will not synchronize.

The above interpretation of the chimera state seems to have a wide range of applicability. Other than the results presented here, for 50 ≤ *N* ≤ 800, we have also calculated the conditional Lyapunov exponents for the various couplings described in ref.^16^. In all cases, the driving chimera state can only synchronize the response system, when all the conditional Lyapunov exponents become negative. Synchronization only occurs when at least all the incoherent oscillators are used as the drive. We have also related our present analysis to the characteristic distribution of finite-time Lyapunov exponents for chimera states, described previously^16^.

### Finite-time distributions of (conditional) Lyapunov exponents

Before discussing the distributions, we emphasize once more that Pecora and Carroll’s conditional Lyapunov exponents are independent of the response system. So are the finite-time conditional Lyapunov exponents. For conceptual clarity, therefore, we emphasize here that the distributions which follow are independent of whether the ‘drive’ is, or is not, connected to the response system.

**Figure 4:**
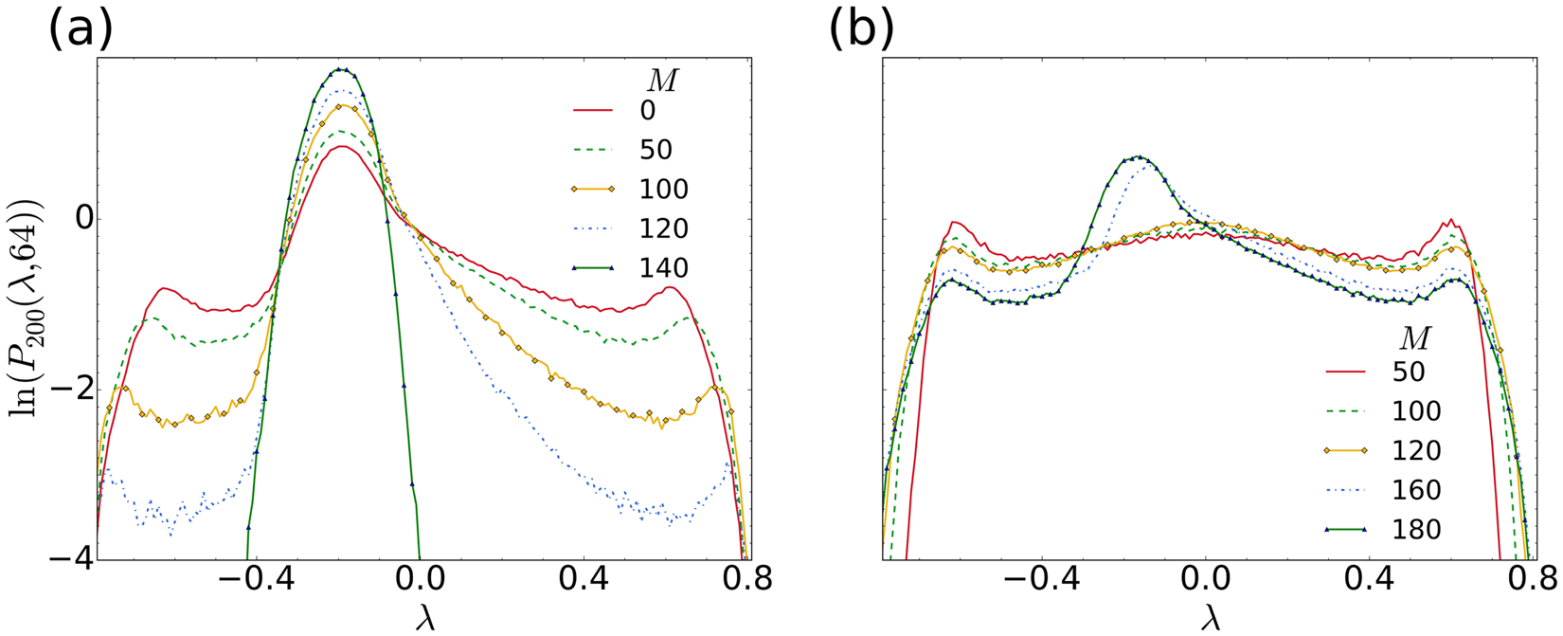
Distributions of finite-time Lyapunov exponents. We consider the incoherent-centred drive configuration in a system with *N* = 200 oscillators. All other parameters are the same as before. In (**a**), we show how the distributions of finite-time Lyapunov exponents evolve away from the characteristic shape that was described in our previous work^16^. This characteristic shape corresponds to the *M* = 0 case, for the full spectrum of finite-time Lyapunov exponents. As *M* increases the distributions obtained from the conditional finite-time Lyapunov exponents tend toward a Gaussian that is centred on the main peak of the characteristic distribution (*M* = 140). This Gaussian corresponds to all the degrees of freedom associated with the coherent group, made up of the 60 remaining oscillators. In (**b**) the corresponding distributions are shown, when the finite-time Lyapunov exponents are projected onto the subspaces spanned by the variables corresponding to the incoherent oscillators. Here we observe a reduction in the central main peak of the characteristic distribution (shown in (**a**) for *M* = 0), as *M* increases. When *M* becomes close to or equal to *N*, the characteristic distribution associated with the full spectrum of *N* finite-time Lyapunov exponents is recovered. This can be seen by comparing the *M* = 180 case in (**b**) with the *M* = 0 case in (**a**).

In Fig. 4, we make use of the incoherent-centred drive configuration from Fig. 1(a). Here we see an example of how the shape of the characteristic distribution evolves as the full spectrum of finite-time Lyapunov exponents is sequentially projected onto smaller and smaller subsets of the (a) coherent and (b) incoherent subsets of the dynamical variables.

We see that, as *M* increases up to the threshold of approximately 0.7 *N* = 140, the intensity of the main central peak in the characteristic distribution is reduced. This reduction occurs as more and more incoherent oscillators are being removed (projected out) from the dynamics; their effect after projection, only being felt conditionally through the nonlocal coupling that exists in this system. In the previous work^16^ it was suggested that the central peak seen in the characteristic distribution of a chimera is related to degrees of freedom corresponding to the coherent domain. In Fig. 4 we see that this interpretation is consistent with conceptual subdivision of the system into a drive-response system. The present analysis offers a more precise interpretation of the central peak in the characteristic distribution: the central peak directly corresponds to the dynamics of the coherent domain, and is indirectly (conditionally) dependent, through the nonlocal coupling, on the incoherent domain.

### Smallest chimera states

**Figure 5:**
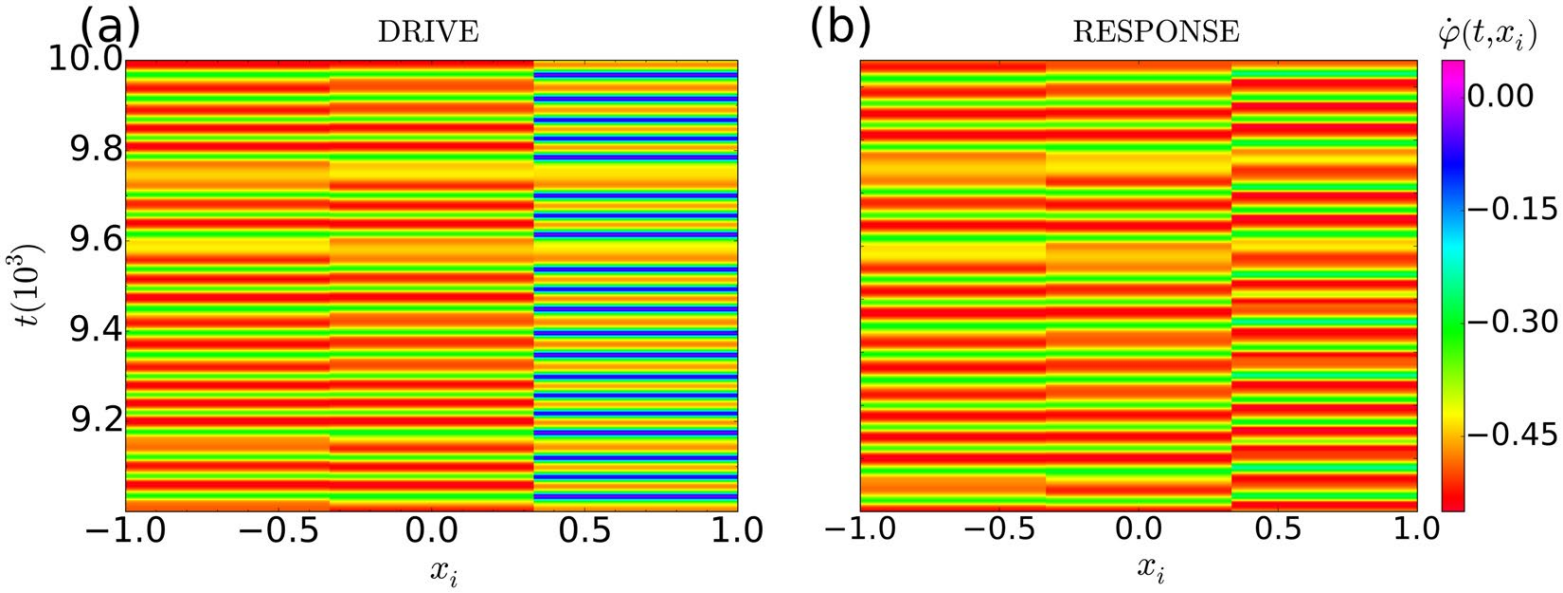
Frequency-time plots for a chaotic chimera. For this ‘smallest’ system, described in ref.^24^, there are only three oscillators; however, the additional inertial terms make the system six-dimensional. Here the system on the right (**b**) is being driven by the first two oscillators from the system on the left (**a**). These two systems do not synchronize because the two conditional Lyapunov exponents, 0.0635 and −0.164, are not both negative. The initial conditions in this simulation were *φ*_1_ = *φ*_2_ = *φ*_3_ = 0, ${\dot{\phi }}_{1}=0.37$, ${\dot{\phi }}_{2}=-0.5$, ${\dot{\phi }}_{3}=0.49$, for the drive, and ${\theta }_{3}={\dot{\theta }}_{3}=0$, for the response. Parameters: *α* = 1.765, *μ* = 0.06, *m* = 1.0, and *ε* = 0.1.

Interestingly, for the so-called ‘smallest’ chimera states^24^, for which there are only three globally coupled oscillators, with an added inertial term, we could not get the two systems to synchronize for any of the chaotic chimera states reported in ref.^24^. An instructive example of this ‘smallest’ system is shown in Fig. 5. The failure of the two systems to synchronize, in this case, points to a fundamental difference between the chaotic chimeras observed in the ‘smallest’ system, compared to the classic chimera states that were discussed in ref.^16^. The chimera nature of this chaotic chimera is due to the fact that two of the average frequencies of the three oscillators are the same, and different from the third: −0.4422, −0.4422, −0.3114. At the same time, both the drive and response systems are chaotic, having very similar Lyapunove exponents: 0.01743, 0.0, −0.00011, −0.05496, −0.10111, −0.16438. The crucial difference between this chaotic chimera, and a classic chimera state, is the fact that the free oscillator in the response system oscillates chaotically, as can be seen in the far right column of Fig. 5(b). Thus, one can say that the coupling between the free oscillator and the two driving oscillators, is not order producing, as we observed previously for the incoherent drive configuration in the classic chimera.

## Discussion

The drive-response theory of Pecora and Carroll^21,22^ shows how two chaotic systems can synchronize, if they are dynamically coupled the right way. Here, we have used their theory to bring out the stability of the frustrated state over the fully synchronized state. The character of the frustrated state is better discovered in this new light, as we find out about the relative size of the coherent and incoherent sets. We gain insight into the chaotic nature of the frustrated state. This chaotic character, however, is reduced as it happens in a collective state, within the chimera. A measure of this reduction could be estimated, using the Kaplan-Yorke conjecture^25^. This conjecture equates a dimension based on the Lyapunov exponents with the geometric dimension of the attracting set. The latter is the entropy or information dimension, pointing to the interrelations within the incoherent set, and among the set of oscillators as a whole. With many positive, but near zero Lyapunov exponents, this is found to be close to half the number of incoherent oscillators, and scaling in general as 0.35 *N*, which is less than if the chaotic set were on its own. For instance, this gives 0.7 for the Henon map, and 1.05 for the Lorentz map, as compared to 1.26 and 2.07 for their actual Kaplan-Yorke dimensions, respectively. We therefore believe our analysis has provided a better understanding of the formation of the chimera, and how it is supported by its frustrated structure.

Our results can also be interpreted in the language of information theory, where the Lyapunov exponents measure the rate at which the system processes create or destroy information^26^. Analogous to the order produced by the flow of thermal energy in non-equilibrium systems, the coherent group in the chimera seems to destroy the dynamic information produced by the incoherent group, thus creating more order than would otherwise exist in the chaotic system. It would be very interesting to make a more quantitative study of the internal flow of dynamic information, perhaps in terms of entropy considerations^27^, within chimera states.

The stability of the chimera state indicates an inner correlation between the incoherent and coherent subsets of oscillators, which maintains the chimera as it is defined. Previous work along the same theme as we have presented here; namely, coupling two interacting populations of oscillators, has shown how the stability is controlled by the coupling strength, where the chimera emerges as the more stable state^28,29^. The stable state is interpreted as a result of two competing synchronization patterns^29^, leading to the more stable, yet frustrated, state.

Finally, it remains to investigate the relevance, to chimera states, of the numerous developments that have taken place since Pecora and Carroll’s original work^21^. Some of these developments have been discussed in their recent review^23^, which does not mention chimera states.

## Methods

### Numerical integration of Eqs (1) and (2)

The driving is achieved by evolving both systems simultaneously, and ensuring that the components being driven are overwritten by the corresponding drive components in each call to the function returning the derivatives, ${\dot{\theta }}_{k}$. For the numerical integration, we used Python’s dopri5 routine, imported from the module scipy.integrate.ode. This is an explicit Runge-Kutta method of order (4)5, with adaptive step control and dense output^30^. We set the relative and absolute error tolerances to 10^−14^. Since asymmetric solutions to the nonlocally coupled Kuramoto-Sakaguchi model are known to exhibit chaotic fluctuations of their position along the unit circle, particularly at low values of *N*^14^, we mostly calculate the symmetric numerical solution of the drive system, for the cases when *N* ≤ 400. By doing so we avoid having to keep track of which drive oscillators belong to the coherent and incoherent groups, at any instant in time. Our calculations for asymmetric drives at *N* = 800, where the position of the incoherent (and coherent) region is stationary over much larger time scales than used here, confirm that there are no essential differences between symmetric and asymmetric driving.

### Calculation of the Lyapunov exponents

We have calculated the Lyapunov exponents by using the standard (WSSV) code given in Wolf *et al*.^26^, modified according to the method developed by He *et al*.^31^, for the conditional Lyapunov exponents. We performed the Gram-Schmidt orthogonalization of the frame vectors every 10 dimensionless time units, and allowed a transient time of 1000. The Lyapunov exponents were averaged between 1000 and 11000 time units, which is a sufficiently long time to ensure convergence of the largest exponent to within a range of ±0.1% of its average value, during the last 1000 time units of the calculation.

The distributions of finite-time Lyapunov exponents were calculated as in the previous work^16^. To obtain the distributions for the finite-time, conditional exponents we simply set to zero the relevant rows and columns of the Jacobian matrix for the drive system, as explained in the method by He *et al*.^31^. The notation used for the distributions in Fig. 5 is as before. For example, *P*_200_(*λ*, 64) denotes the distribution of exponents (or conditional exponents), for *N* = 200 oscillators, averaged over 64 samples. As in the previous work^16^, our sample time interval is Δ*t* = 1/128. Thus, the finite-time exponents have been averaged over 0.5 dimensionless time units. Each distribution contains 10000 samples, recorded after the usual transient time of 1000.

### Data availability

The datasets generated and analysed during the current study are available from the corresponding author on reasonable request.
